# The Genes Involved in Dentinogenesis

**DOI:** 10.1080/15476278.2021.2022373

**Published:** 2022-01-13

**Authors:** Shuang Chen, Han Xie, Shouliang Zhao, Shuai Wang, Xiaoling Wei, Shangfeng Liu

**Affiliations:** a Shanghai Key Laboratory of Craniomaxillofacial Development and Diseases, Shanghai Stomatological Hospital, Fudan University, Shanghai, P. R. China; b Department of Prosthodontics, Shanghai Stomatological Hospital, Fudan University, Shanghai, P. R. China; c Department of Stomatology, Huashan Hospital, Fudan University, Shanghai, P. R. China; d Department of Endodontics, Shanghai Stomatological Hospital, Fudan University, Shanghai, P. R. China

**Keywords:** dentinogenesis, odontoblasts, Gene, signaling pathway

## Abstract

The development and repair of dentin are strictly regulated by hundreds of genes. Abnormal dentin development is directly caused by gene mutations and dysregulation. Understanding and mastering this signal network is of great significance to the study of tooth development, tissue regeneration, aging, and repair and the treatment of dental diseases. It is necessary to understand the formation and repair mechanism of dentin in order to better treat the dentin lesions caused by various abnormal properties, whether it is to explore the reasons for the formation of dentin defects or to develop clinical drugs to strengthen the method of repairing dentin. Molecular biology of genes related to dentin development and repair are the most important basis for future research.

## Introduction

Dentin constitutes the main body of the tooth and is located in the inner layer of enamel and cementum, the side wall of the pulp cavity and root canal. Dentin acts as a barrier to prevent exposure of the dental pulp, reduce the conduction of hot and cold stimuli to the pulp, and protect the living pulp. In addition, it also provides a hard tissue foundation for later dental restoration.

Odontoblasts are the only known cells that form dentin, and they are involved in the synthesis of primary, secondary, and reactionary dentin. A recent study showed that odontoblasts activate the innate immune response in the pulp.^1^ Although the function of odontoblasts is unique, studies have not advanced new information likely because they are end-stage differentiated cells that are difficult to isolate and culture. To study the dentin formation process, it would be necessary to study gene regulation during the development and migration of odontoblasts.

In this review, we present information on 300 genes involved in dentinogenesis (Table 1) and to date, the related regulatory processes of dentinogenesis has not been fully elucidated. However, new signaling molecules and transcription factors have recently been identified and shown to constitute a complex signaling network that participates in dentin formation. Understanding and mastering this signal network is of great significance to the study of tooth development, tissue regeneration, aging, repair and the treatment of dental diseases.

**Table 1.** The genes involved in dentinogenesis

Pathway	Gene	Main function in dentinogenesis
Extracellular matrix proteins and related	COL1A1/2, COL2A1, COL4A1/2/3/4/5/6	The COL1A1/2 ^2,3^ and COL2A14 mutation results in dentinogenesis imperfecta; Type IV collagen alpha subunits occur during molar germ development and that these changes are essential for molar morphogenesis and cytodifferentiation.^5^
	DSPP, DPP, DSP, DGP	Dentin hypoplasia or dysplasia. It is an important marker of odontoblast.^6^
	DLX3	Deletion of Dlx3 leads to major dentin defects through down-regulation of Dspp.^7^
	TM14	Mainly expressed in odontoblasts,and participates in the formation and mineralization of dentin.^8^
	BSP	As the crystal nucleus, promotes the crystal formation, and participate in dentinogenesis.^9^
	Dmp1/2/3/4	Dmp1/2 participates in the differentiation and dentin formation of odontoblasts.^10,11^ Rat Dmp3 is a compound protein of rat DSP and phosphophoryn.^12^ Dmp4 forms and affects the growth and arrangement of hydroxyapatite crystals, and promotes the differentiation of osteoblasts, ameloblasts and odontoblasts.^13^
	EMILIN-1/2/3	EMILIN-1 and −2 staining appears to increase in the pre-dentin and in the ECM surrounding odontoblasts. EMILIN-3 was significantly increased in inflamed odontoblasts.^14^
	SSUH2	Decrease of collagen in the teeth of mice, which makes the dentin mineralization abnormal.^15^
	GRP78	SSUH2 binds to the Dank domain of GRP78 through DanJ, which affects the transport of collagen and DMP1, there by affecting the mineralization of teeth.^16^
	Sulf1/2	Sulf1/Sulf2 double null mutant mice exhibit a thin dentin matrix and short roots.^17^
	MMP1/2/7/8/9/10/11/13/14/15/16/17/19/20/23/24/25	MMP1 regulate tooth agenesis; MMP2 makes multiple cleavages near the DSP C terminus, releasing larger forms of DGP; MMP8 may be involved in the alteration of dentin matrix during the development of human and rat tooth germ; MMP9 is important for tooth development and DSP is a target of MMP9 during dentinogenesis; MMP14 functions in tooth root formation, dentinogenesis, and tooth eruption; MMP-20 cleaves DSP-DGP to generate DSP and DGP; MMP1, 2, 9, 10, 11, 13, 14, 15, 16, 17, 19, 20, 23, 24 and 25 expressed in odontoblasts.^18–20^
	TIMP1/2/3	Expression was detected in odontoblasts.^21^
	Osteocalcin	Both crown and root odontoblasts and dentin stained for Osteocalcin.^20^
	Osteonectin	Osteonectin was present mostly in the nonmineralized predentin.^22^
	MEPE	It plays an important role in the formation of dentinal tubules and pulpal homeostasis.^23^
	PHEX	A decrease in PHEX expression could suppress dentin formation.^24^
	Versican, Decorin, Appican, Biglycan, Glypican, Syndecan-1/3	Decorin, biglycan, syndecan-1 and syndecan-3 showed gene expressions overlapping with OASIS. Especially the expression pattern of decorin and syndecan-3 coincided temporally and spatially exactly with that of OASIS.^25^
	Tenascin	Association with dentinal tubules, particularly prominent in the tooth crown.^26^
	Tuftelin	Tuftelin could be secreted by preodontoblast cells and preameloblast cells.^27^
	Reelin	Reelin may promote adhesion between dental nerve endings and odontoblasts.^28,29^
	S100-A7	S100-A7 released from dentin by MMP20 might play a key role in dentin pulp regeneration.^30^
Mineralization related pathway	Bono1	Expressed in functional odontoblasts and was associated with regions of matrix mineralization.^31^
	Runx2	Participation in dentin formation, mineralization, and development of odontoblasts.^32^
	Msx2	Msx2 expressed in the odontoblast.^33^
	PAX9	Tooth agenesis.^34^
	Opn	Essential for type I collagen secretion in reparative dentin.^35^
	Vdr	Vitamin D receptors deficiency compromises dentin maturation.^36^
	Smpd3	Deletion of smpd3 induces dentinogenesis imperfecta in mice.^37^
	Osad	Osad may play an important role during tooth development and biomineralization of dentin.^38^
	SOST	Sclerostin deficiency hastened reparative dentinogenesis after pulp injury.^39^
	ANKH	Direct mineralization in cementum and likely other mineralized tissues.^40^
	OSX	Involved in the differentiation, maturation and intercellular signal transduction of odontoblasts.^41^
	SMOC2	Dentin dysplasia, small teeth, missing teeth.^42^
	CCN2	Associated with reparative dentinogenesis.^43^
	PACE4	PACE4 plays a crucial role in dentinogenesis, especially via the activation of BMPs.^44^
	Alp/Alpl	Tooth mineralization.^45^
	RANK/RANKL	Associated with delayed permanent tooth emergence and tooth development.^46^
	OPG	Resulted in reduced mineralization.^46^
Wnt Signaling Pathway	Timp1	Play crucial roles in reactivation of immature pulp cells for tertiary dentinogenesis.^47^
	CTNNB1	Tooth agenesis.^48^
	APC	Supernumerary tooth; Odontoma.^49^
	Kremen1	Ectodermal dysplasia including oligodontia.^50^
	LRP6	Oligodontia.^51^
	Axin2	Axin2-expressing cells differentiate into new odontoblast-like cells that secrete reparative dentine.^52^
	Ctbp1	Hypotonia.^53^
	SFRP2	Key factor in maintaining cell survival following dentinogenic commitment.^54^
	Lef1	Play a key role in odontoblast differentiation through regulating Dspp expression.^55^
	Wnt1/2/3a/4/5a/5b/6/7a/7b/8a/8b/9b/10a/10b/11/13/14/16	Tooth agenesis.^56,57^ mediation of dentinogenesis.^58,59^
	VPS4B	Regulate tooth development.^60^
Shh Signaling Pathway	Shh	Shh regulates growth and determines the shape of the tooth.^61^
	Ptch1/2	Regulate teeth stem cell maintenance and differentiation.^62^
	MSX1/2	Msx1 and Msx2 play a major role in tooth formation.^63^
	Gli1/2/3	Gli1^+^ cells in mature teeth appear to contribute to the regeneration of dental pulp and periodontal tissues. Gli2 mutants were found to have abnormal development of maxillary incisors, whereas Gli3 mutants had no major tooth abnormalities. Gli2/Gli3 double homozygous mutants did not develop any normal teeth.^64,65^
	Sufu	Modulating the tooth germ morphogenesis during the bud-to-cap stage transition.^61^
	Nfic	Nfic has an essential role in tooth root formation.^66^
	Kif3a	Kif3a-deficient mice results in tooth dysplasia.^67^
TGFβ Signaling Pathway	TGF-β	Matrix formation and pulpal obliteration.^68^
	Activin βA	Activin βA by follistatin may allow odontoblast terminal differentiation to occur.^69,70^
	Follistatin	Activin-follistatin system regulates odontoblast differentiation during tooth development.^69^
	Islet1	Exclusively expressed in epithelial cells of the developing incisors during odontogenesis.^71^
	Ectodin	Ectodin inhibited the activity of BMP2, BMP4, BMP6, and BMP7.^72^
	BMP1/2/3/4/5/7/9	Play a crucial role in organogenesis, including tooth development.^73,74^ Participates in the process of cell differentiation, mineralization and dentinogenesis.^75^
	KLF4	Klf4 promotes dentinogenesis and odontoblastic differentiation via modulation of TGF-β signaling pathway and interaction with histone acetylation.^22^
	p300, HDAC3	p300- and HDAC3-regulated odontoblast differentiation through upregulating histone acetylation.^76^
Notch Signaling Pathway	Notch 1/2/3	Upregulation of Notch signaling pathway after tooth injury.^77^
	TSPEAR	Mutations in TSPEAR, encoding a regulator of Notch signaling, affect tooth morphogenesis.^78^
	DLK1	Inhibited the odontoblastic differentiation of hDPSCs.^79^
TNF Signaling Pathway	Eda, Edar, Edaradd	Tooth agenesis.^80^
	Traf6	Regulate cuspal morphogenesis.^81^
	TNFRSF19	Expressed in an overlapping domain with Edar in the tooth.^82^
Ion channel	Nav1.1/1.2/1.3/1.4/1.5/1.6/1.7/1.8/1.9	Nine voltage-gated sodium channels are all expressed in odontoblasts, and their expression location depends on the tooth position and tooth maturity. Related to tooth sensitivity.^83^
	TRPC1/2/3/4/5/6/7	TRPC1-7 belongs to the calcium channel family and were mainly expressed in odontoblasts.^84^
	CLCN1/2/3/4/5/6/7	Regulated tooth development through effects on cell proliferation and cell cycle signal pathway.^85^
	TRPM3/7/8	Both odontoblasts and dental pulp cells express TRPM channels in rat, mouse and human tooth.^86^
	Piezo1/2	Expressed in odontoblasts.^87^
	TRPV1/2//3/4	TRPV1/2//3/4 channels expressed in odontoblasts.^88^
	P2X3/4/5/7, P2Y1 /2/4/6/11/12/13/14	Extracellular ATP activates P2 receptors and downstream signaling events that induce cell odontogenic differentiation.^89^
	AQP4/5	AQP4 and AQP5 immunostaining was observed in the odontoblasts and their processes.^90^
	AHR	Tooth mineralization.^91^
Growth factor	FGF1/2/3/4/7/8/9/10/11/12/13/15/17/20, FGFR1/2/3	Function in dentinogenesis.^92–96^
	COUP-TFII	Matrix mineralization in odontoblast-lineage cells.^97^
	β2AR	The sympathetic nervous system decreases tertiary dentin formation via β2AR.^98^
	PTH/PTHrP/PTH1R	Essential signal in the formation of the eruption pathway.^99^
	IGF-1	IGF-1 can weaken odontogenic differentiation and dentinogenesis capability.^100^
	EXT-1	Function in the dentin formation.^101^
	Oxytocin	Promote odontoblast-like cell differentiation, resulting in increased dentin formation.^102^
	IGFBP5/6/7	IGFBP5/6/7 may play independent and redundant regulatory roles in late-stage odontogenesis.^102^
Stress response	HSP25/70	Hsp25 is involved in reinforcement of the cell layer following cell movement during odontogenesis. Hsp70 might play an important role during reparative dentin formation.^103^
	Ape1	Promote the odontogenic differentiation capacity.^104^
	DRP1	DRP1 inhibition accelerates dentin formation through mitochondrial elongation and activation.^105^
	MTCO2	Age-related changes marker in odontoblasts.^106^
	PPARα/γ	Active PPARα signaling is required to achieve normal mineralization of molar enamel. PPARγ in pulp cells increases cell viability, odontoblastic differentiation, and dentin mineralization under oxidative stress.^107,108^
	LAMP2	Lysosomal (LAMP2) markers, ageing and stress related in odontoblasts.^106^
	SIRT4	Sirt4 knockdown resulted in reduced odontogenic differentiation and mineralization.^109^
Nervous system related pathway	GDNF	Function in odontoblasts develop, differentiate, and the matrix and predentin layers formation.^110^
	Nestin	The original odontoblasts may differently regulate Nestin expression.^111^
	NRG-1, ErbB3/4	NRG-1 and the receptors ErbB3/4 are expressed locally during rodent tooth development.^112^
	Lhx8	Regulates dentin development and regeneration.^113^
Cell junction related genes	OCLN, CLDN1, Zo1/2	Play an important role in the differentiation of odontoblasts.^114^
	CNRs, Pcdh-γ, Reelin	Related to both morphogenesis and cell differentiation events.^28^
	E-cadherin, P-cadherin	Differential and specific roles for E-cadherin and P-cadherin during the morphogenesis.^115^
	Connexin26/32/43	Expressed in odontoblasts.^116^
Bile secretion pathway	SLC2A1, SLC4A4, ADCY5, ATP1B1, SLC10A1, ABCC3	Expressed in tooth germ odontoblasts.^117^
Other related genes	IFT140	IFT140 is essential in promoting dentin formation and reparation.^118^
	EphrinB1, EphB2	Regulates odontogenic differentiation and the early stages of tooth injury.^119^
	KDM1A, KDM5A	KDM1A have function for the dentinogenesis.^120^ Inhibits the odontogenic differentiation and plays an important role in reparative dentinogenesis.^121^
	PrP	Odontoblasts showed prominent staining for PrP at levels comparable to those of nerve fibers.^122^
	HtrA1	HtrA1 might positively regulate odontoblastic differentiation.^123^
	PP1	PP1 might be a potent regulator of odontoblastic differentiation and dentinogenesis.^124^
	Sema3A	Play an important role in dentin regeneration via canonical Wnt/β-catenin signaling.^125^
	CPNE7	CPNE7 induced odontoblast differentiation in vitro and promoted dentin formation in vivo.^126^
	MAP1B	MAP1B could be involved in the terminal differentiation of odontoblasts.^127^
	Phospho1	Function in the early mineralization of mantle dentin.^128^
	Midkine	Midkine promotes odontoblast-like differentiation and tertiary dentin formation.^129^
	Cdc42	Is particularly required for cell survival and tooth morphogenesis.^130^
	Sp1/3/6/7	Sp1 promotes the odontoblastic differentiation and mineralization of dental papilla cells^131^; Sp3 is essential for post-natal survival and late tooth development^132^; Sp6 has been found to present striking dental abnormalities^133^; Sp7 is required for proliferation and differentiation of odontoblasts.^134^
	Zeb1	Promoted odontoblast differentiation in the early stage.^135^
	Fubp1	Plays a modulating role during dentinogenesis.^136^
	GATA4	Important for root formation and odontoblast polarity.^137^
	ADAMTS2	Mutation in ADAMTS2 causes multiple tooth agenesis and focal dysplastic dentin defects.^138^
	Trps1	Trps1 functions as a repressor of later stages of dentinogenesis.^139^
	OASIS	Play an important role in the differentiation of the odontoblast.^25^
	Hand2	Essential for odontoblasts cells during development.^140^
	RICK	Regulates odontogenic differentiation of dental pulp stem cells.^141^
	WWP2	Promotes Odontoblastic Differentiation.^142^
	TANGO1	Severe dentinogenesis imperfecta.^143^
	Mdm2	Promotes the odontoblast-like differentiation.
	Glut1/2/4	Dentinogenesis.^32,144^
	MTOR	Reparative dentinogenesis.^32^
	CB1	Enhance the dentinogenic differentiation ability.^145^
	Parp-1	Involved in the regulation of continuous dentinogenesis in the incisors at an advanced age.^146^
	Usp34	USP34-dependent deubiquitination is critical for root morphogenesis by stabilizing NFIC.^147,148^
	mTORC1	mTORC1 involed in odontoblast proliferation and mineralization.148

### Extracellular matrix proteins and related

Extracellular matrix (ECM) proteins could be directly involved in the construction of dentin because the mutation, deletion, or abnormal regulation of these proteins often leads to serious dentin-related diseases. Presently, all ECM proteins have been identified; however, their functional regulation still needs to be comprehensively studied. Furthermore, new regulatory mechanisms and key regulatory molecules are still being discovered.

Osteogenesis imperfecta (OI) with systemic skeletal dysplasia is a genetic systemic connective tissue disorder involving the bone, dentin, sclera, ear, blood vessels, skin, and other tissues. Type I collagen (*COL1*) gene mutations(COL1A1 and COL1A2)^2,3,4^ are the main cause of this disease.

Bone and dentin have similar mineralization processes, during which osteoblasts and odontoblasts first secrete unmineralized matrices termed osteoid and anterior dentin, respectively. The organic component of osteoid and anterior dentin consists of an ECM primarily composed of COL1 and several non-collagen proteins that play essential roles in the mineralization of collagen fibers during the conversion of osteoid and anterior dentin to bone and dentin, respectively.^9^ Bone- and odontoblast-expressed gene 1 (*Bono1*) is a gene expressed in functional odontoblasts that is associated with regions of matrix mineralization.^31^ BSP may play an important role in the formation and mineralization during dentinogenesis.^9^

Dentin sialoprotein (DSP) and dentin phosphoprotein (DPP) are encoded by dentin sialophosphoprotein (Dspp) and its deficiency may lead to dentin hypoplasia or dysplasia and opalescent, shell, and abnormal deciduous teeth with short roots. Therefore, Dspp is an important marker of odontoblasts^6^ and is the first direct target of distal-less homeobox 3 (Dlx3), which was identified in odontoblasts.^7^

In addition, TM14 has significant roles in the differentiation and maintenance of odontoblasts, and in dentin formation.^8^ As a nucleus, bone sialoprotein (BSP) promotes crystal formation and in developing teeth it is well documented to form acellular cementum and periodontal attachments. However the expression and function of BSP in odontoblasts and dentin are uncertain.^9^ Dentin matrix acidic phosphoprotein 1/2 (Dmp1/2) participates in odontoblast differentiation and dentin formation.^10,11^ Rat Dmp3 is a compound protein of rat DSP and phosphophoryn.^12^ The formation of Dmp4 affects the growth and arrangement of hydroxyapatite crystals and promotes the differentiation of osteoblasts, ameloblasts, and odontoblasts.^13^

Matrix metalloproteinase 1 (MMP1) regulates tooth agenesis; MMP2 causes multiple cleavages near the DSP C terminus, releasing larger forms of DGP; and MMP8 may be involved in the alteration of dentin matrix during the development of human and rat tooth germ. In addition, MMP9 is important for tooth development and targets DSP during dentinogenesis, whereas MMP14 functions in tooth root formation, dentinogenesis, and tooth eruption. Furthermore, MMP20 cleaves DSP-DGP to generate DSP and DGP, and MMP1, 2, 9, 10, 11, 13, 14, 15, 16, 17, 19, 20, 23, 24 and 25 are expressed in odontoblasts.^18–20^

The dentin organic matrix is secreted by odontoblasts during dentinogenesis and mineralized in the predentin area.^149^ In dental attrition, hardened molds and highly mineralized layers were detected in sclerotic dentin. During the process of sclerotic dentin formation and long-term aging, the MMP content in dentin changes.^150^ The process of dentinogenesis is similar to bone formation and many genes are involved in both processes.

Runt-related transcription factor 2 (Runx2) participates in dentin formation, mineralization, and the development of odontoblasts.^32^ Runx2 is also essential for the differentiation of osteoblasts and odontoblasts, and it regulates the expression of numerous bone- and tooth-related genes. Runx2 determines the lineage of osteoblasts and odontoblasts in the mesenchymal cells. Osterix (*OSX*), a downstream gene of Runx2 in the osteoblast differentiation signaling pathway, is involved in the differentiation, maturation, and intercellular signal transduction of odontoblasts.^41^ Osteoprotegerin (OPG) is expressed in both the thickened and bud epithelium, as well as in combination with receptor activator of nuclear factor-κΒ (RANK) in both the enamel and papillary stroma. Although RANK ligand (RANKL) was not detected in the tooth epithelium or mesenchyme, it was expressed in pre-osteogenic mesenchymal cells near the developing tooth germ.^46^

### Mineralization related pathway

Followed by the pre-section, we reviewed some key proteins related with odontoblasic differentiation or dentinogenesis. In further, the mineralization related pathways those could upregulate the mentioned proteins also can affect odontoblastic differentiation.

### Wnt signaling pathway

The Wnt signaling pathway could currently be considered the most thoroughly studied pathway and drug research related to dentin regeneration has also achieved significant breakthroughs recently. However, the involvement of the Wnt non-classical signaling pathway in the dentin formation process has not been elucidated. This pathway plays a crucial role in the reactivation of immature pulp cells for tertiary dentinogenesis.^47^ TIMP1 plays a crucial role in the formation of tertiary dentin.

In addition, cavity preparation may activate the Wnt/β-catenin pathway.^47^ Wnt signaling is essential to both the epithelium and stroma, whereas heterologous inactivation of β-catenin leads to stagnation of early tooth germ development. The elimination of mesenchymal-specific β-catenin stagnated tooth germ development, which indicates that Wnt signaling plays a role in the transition from the bud stage to the cap stage during tooth germ mesenchymal development. ^50^

Wnt10a was shown to be strongly expressed in the odontoblast layer and was specifically expressed in the secretory odontoblasts co-expressed with DSPP.^56^ The role of the Wnt/β-catenin signaling pathway in odontoblast differentiation and dentin formation is mediated through its component proteins.^56^ Phenotype-related diseases are sometimes caused by paralog genes, which may explain the dental abnormalities in patients with Wnt10a and Wnt10b mutations.^57^ Wnt signaling is essential for normal tooth development and its persistent activation leads to the continuous renewal of teeth and supernumerary teeth, whereas its inhibition stagnates tooth development. Abnormal Wnt signaling has been shown to cause a variety of human developmental disorders ranging from the lack of a tooth to life-threatening cancer.^57^

### SHH signaling pathway

Sonic hedgehog signaling molecule (SHH) has been shown to regulate dentin formation mainly during embryo development. GLI family zinc finger 1 (Gli1)-positive cells in mature teeth have been found to have stem cell properties that contribute to the regeneration of pulp and periodontal tissue. SHH, a secreted protein that plays a significant role in mammalian embryogenesis, regulates growth and determines the tooth shape. Dental epithelial SHH regulates tooth morphogenesis through epithelial mesenchymal signal transduction. Many studies have analyzed the function of SHH signaling in different stages of tooth development, and have reported that it regulates the formation of various tooth components, including the enamel, dentin, cementum, and other soft tissues.^61^ SHH is mainly expressed in the dental epithelium during tooth development.^64^

The stratum intermedium is a highly dynamic and SHH-expressing structure that undergoes marked and transient changes in the histological organization and phenotype during odontogenesis. The stratum intermedium is involved in the development of the tooth germ, which has not been previously reported.^151^ This delicate cell group has undergone an amazing process of evolution and degradation, which is closely related to the process of development from the dental cusp to the cervix. SHH is one of the signaling molecules for which the intermediate layer may play a role in mediated its function.^151^ The primary cilia are essential for the integration of Wnt and Hh signals and in their functional absence, SHH signals decrease in the dental stroma, whereas those of Wnt increase in the dental mesenchyme.^67^

### TGFβ signaling pathway

The transforming growth factor β (TGFβ) signaling pathway is mainly involved in the differentiation and regulation of odontoblasts and includes the BMP family of molecules, which showed the most remarkable effects.

The TGFβ family plays an important role in matrix formation and pulpal obliteration, especially in pulp-dentin pathophysiology. TGFβ induces the secretion of ECM components related to primary and tertiary dentin formation. TGFβ isoforms are also expressed by mature odontoblasts, leading to the isolation of these growth factors in the dentin matrix. This process provides a matrix-associated TGFβ library that can be released in matrix changes associated with caries injury or trauma.^68^ BMPs are signaling molecules secreted by the TGF superfamily and more than 30 types are known to regulate embryonic development in almost all tissues and organs of all animals and Fine-tuning of BMP is very important for its various functions.^72^

### Notch signaling pathway

The Notch signaling molecule expression and activation are critical not only for the development of tooth embryos, but also for the regeneration of damaged tissues of adult teeth. Notch is an important regulator of stem cell fate and can induce cell proliferation and differentiation. There is a close relationship between dental pulp mesenchymal cells and neovascularization in dental pulp diseases and the Notch signaling pathway is upregulated after tooth injury.^77^

### TNF signaling pathway

The specific role of the tumor necrosis factor (TNF) family of molecules identified in tooth morphogenesis suggests that this pathway plays an important role in the development and evolution of the tooth number and shape.^82^ Ectodysplasin A (EDA), a member of the TNF superfamily, and its receptor, EDAR, are an essential part of ectodermal organ development. Analysis of their expression patterns and mutant phenotypes has shown that they may participate in signal transduction between different epithelial cells during hair and tooth development in mice.^82^

### Ion channel

Ion channels play an important role in various kinds of proprioception, pain, and conduction of hot and cold stimuli in teeth, as well as in calcium transport, deposition, and loss of teeth.

Voltage- and ligand-gated ion channels play a significant role in toothaches. The sensory fibers of the trigeminal nerve possess different types of voltage-gated ion channels expressed in common nerve cells and odontoblasts. Previous studies have shown that small molecules such as 5 ‘- adenosine triphosphate (ATP) and its ion receptor of the P2X family play an essential role in the sensory system of mediating toothaches. ^88^

### Growth factor

It is involved in the development of teeth and the formation of secondary and reactionary dentins.

Although there is evidence that tooth morphogenesis and innervation are independent, the role of nerve fibers in tooth development or dentin and enamel formation remains controversial. In our study, the first molar tooth germ neurons of glial -derived neutrotrophic factor (GDNF)-deficient and wild-type mice were almost identical in structure and Schwann cell density.^110^ This suggests that GDNF, similar to other members of the GDNF family such as neurturin, is not involved in guiding or maintaining the neural structure during tooth development or in the survival of Schwann cells.^110^

Among the members of the BMP family, the biological function of BMP 2 in root development has been widely studied and it promotes the differentiation of dental pulp stem cells into odontoblasts. IGF-1 promotes osteogenic differentiation and osteogenesis of bone, but can also reduce its odontogenic differentiation and ability, suggesting that IGF-1 could be as a candidate material for bone tissue engineering. The osteogenic differentiation signaling pathway of stem cells from apical papilla (SCAP) induced by IGF-1 requires further study, whereas IGF-2 appears to preferentially play a role in enamel deposition.^152^

### Stress response

Presently, the molecular regulatory mechanism of the stress response in teeth is not well understood, although it is of great significance to tooth development and the formation of secondary and reactionary dentins. The expression of heat shock protein 25 (Hsp25) in odontoblasts could be considered a stress response, which can be achieved by regulating actin dynamics and programmed cell death. Following the development of dentin and its deposition, the odontoblast retreated from the apex to the incisor end and the pulp space in the middle is reduced, forming pseudo-stratification. This histological finding suggests that odontoblasts undergo mechanical stress due to increase cell density during active dentin formation and this environmental change may induce a strong Hsp25 immune response in these cells.^153^

### Nervous system related pathway

Nerve-related genes involved in the regulation of dengtinogenesis have also been reported. Some studies have shown that during pulp regeneration odontoblast cells are likely derived from Schwann cells in the pulp, but the fine regulatory mechanisms involved are still poorly understood.^110^ The role of nerve fibers in triggering the development of the teeth or in the onset of dentin and enamel formation is still controversial, although evidence suggests that tooth morphogenesis and innervation are independent. In our study, the first molar tooth germ (FMTG) nerves were almost identical in structure and density to the Schwann cells in GDNF-deficient and wild-type mice. This suggests that glial cell-line derived growth factor (GDNF) and neurturin are not involved in guiding or maintaining the structure of nerves in the developing tooth or in the survival of Schwann cells.^110^

### Cell junction related genes

The role of the cell junction in oral development and disease is poorly understood. Occludin (OCLN), claudin-1 (CLDN1), and zonula occludens-1/2 (Zo1/2) play important roles in odontoblast differentiation.^114^ CNRs, protocadherin (Pcdh)-γ, and Reelin are related to both morphogenesis and cell differentiation events.^28^ E-cadherin and P-cadherin have differential and specific roles during morphogenesis^115^ and connexin 43 is expressed in odontoblasts.^116^ The expression patterns of CLDN1, OCLN, ZO-1, and ZO-2 are different. Tight junctions (TJs) of the rat lower incisor odontoblasts may play an important role in the early differentiation of odontoblasts, especially in determining the direction of mineral secretion and establishing the distal membrane domain.^114^

### Bile secretion pathway

Recent studies have shown that this signaling pathway, ostensibly unrelated to teeth, is involved in tooth embryo development, and tooth formation involves strict genetic control procedures.^117^ Therefore, exploring the gene network system regulating tooth development has a very positive practical significance in the study of tooth tissue regeneration and the prevention and treatment of tooth abnormalities. The early bell stage is the initial phase of odontoblast formation and dentin matrix deposition in the tooth development process. RNA sequencing and differential gene analysis of rat tooth germ samples at the cap and early bell stages showed that the bile secretion pathway was the most significantly different between the cap and bell stages among related signaling pathway during development, which mainly included SLC10A1, SLC2A1, SLC4A4, ADCY5, ATP1B1 and ABCC3.^117^

### Other related genes

However, the major signaling pathways involved in dentinogenesis and new genes remain to be discovered and elucidated. For instance, IFT140 is essential in promoting dentin formation and reparation.^118^ Odontoblasts showed prominent staining for PrP at levels comparable to those of nerve fibers.^122^ Glucose supply via GLUT1 might occur before the differentiation of odontoblast-like cells. The transport of glucose via Glut2/Glut4 might contribute to the production of a dentin bridge during wound healing.^32,144^ Mdm2 promotes Odontoblast-like differentiation by Ubiquitinating Dlx3 and p53. It will continue to add and classify, because the number is small and not deep enough.

## Conclusion and perspectives

The development and restoration of dentin is a complicated and strict regulatory process and numerous signaling molecules and transcription factors constitute the complex signaling network mediating dentin formation. Understanding these signal networks is of great significance to the study of tooth development, damage repair, and tissue regeneration and to the treatment of abnormal tooth development. We believe that research in this field should focus on the following points in the future.

### Odontoblasts aging

Odontoblast aging is a field that has rarely been studied in the past decade and similar to other major functional cells, odontoblasts have a process of development, maturation, and senescence. To date, the specific genes involved in odontoblast cell aging are unknown. Odontoblast aging is closely related to dentin regeneration. The autophagy-lysosome system of odontoblasts ensures the renewal of organelles and proteins, thereby extending their lifespan. However, the gradual accumulation of lipofuscin in lysosomes reduces the viability of odontoblasts and the ability of dentin to regeneration.^154,155^ Moreover, the old dentin structure is also different from that of young dentin, and a study on the mechanical behavior of dentin reported that all stiffness, strength, and fatigue properties tested decreased significantly with age.^156^ Scanning electron microscopy revealed that old dentin exhibited more intratubular crystal deposits than the young dentin, which had an 80% lower permeability.^157^ We hope that future research will elucidate the process and molecular mechanisms of odontoblast aging, which could provide strategies to regulate odontoblast aging or reverse the aging process.

### Odontoblasts immune responses

The odontoblast is the first tissue barrier against invasion of the tooth by caries-related pathogens, and is the starting and effective point of the immune response of the tissue to these pathogens. However, the pattern recognition receptors (PRRs) involved in the responses to pathogen-associated molecular patterns (PAMPs) in odontoblasts have not been fully clarified. Therefore, a deeper understanding of the mechanisms underlying PAMP-induced innate immune responses and the role of PRRs in odontoblasts would help the development of strategies to maintain dental pulp tissues in a healthy condition for as long as possible. Furthermore, the enhanced understanding of these processes may lead to the development of novel therapeutic strategies and treatments for pulpitis.

### Dentin development

Currently, although there is considerable information available on the dentin developmental process and related genes, the details of its complex regulatory network are still unclear. Furthermore, repairing dentin abnormalities or defects that have occurred is challenging. Presently, prenatal or preimplantation genetic diagnosis is the most effective method of solving dentin developmental problems. Future further studies and understanding of the regulatory network of dentin development would contribute to enabling the modification adult mutant genes for the benefit of patients by combining modern gene modification techniques such as CRISPR/cas9.

### Epigenetics in dentin development and aging

Recent studies have demonstrated the important role of epigenetics in dentin development and the main epigenetic modifications include DNA methylation, histone acetylation, and methylation.^158^ However, because of the limitations of in vivo and in vitro models and research techniques, epigenetic research on dentin development has only just begun. A considerable amount of research is still required to reveal rules and interventions in developmental abnormalities.

### Dentin regeneration

Dentin regeneration, especially regenerative restoration after dentin injury, requires an understanding of the occurrence and development of dentin, because the two main signaling pathways are very similar. Dentin regeneration requires dental pulp cells and is closely related to dental pulp regeneration.^159^ In addition to previous studies on dentinogenesis, some novel approaches have been explored in the past year. For example, a novel injectable treated dentin hydrogel (TDMH) has been developed for use as a pulp capping material for dentin regeneration.^159^ Histological results showed that TDMH allowed the harvesting of thicker formed dentin than that of Biodentine and mineral trioxide aggregate (MTA).^159^ Another study utilized an amphiphilic synthetic polymeric combined with exosomes derived from both dental pulp stem cells and immortalized murine odontoblasts as dental pulp capping material to generate dentin *in vivo*. After 6 weeks, the exosome group exhibited higher quality formation of the dentin bridge than the group treated with glass-ionomer cement.^154^ The development of nanotechnology has also led to the application of nanomaterials in new strategies in the field of dentin regeneration. Mesoporous bioactive glass/graphene oxide composites have been shown to improved mineral differentiation of human dental pulp stem cells by upregulating the odontogenesis-specific markers DSPP and DMP-1. This observation suggests that this composite may induce stem cell differentiation into odontoblast-like cells and thereby induce dentin formation.

### Physical factors

Some physical factors including ultrasound, static magnetic field(SMF), electric field(EF), and laser irradiation also affect the differentiation of odontoblasts. Ultrasound: In the experimental model of dentin injury without pulp exposure, low-intensity pulsed ultrasound (frequency: 1.5 MHz, 200 μs pulse width, 1 kHz pulse repetition frequency, 30 mW/cm^2^ spatial averaged temporal averaged intensity) treatment of teeth increased calcium ion transport-related protein (Cav1.2, NCX1 and TRPV1) expression. After 14 days, hematoxylin and eosin (H&E) staining showed more significant dentin formation in the pulse treatment group than that in the other groups and the underlying mechanism may involve inflammatory reactions and mechanical effects.^160^

Static magnetic field (SMF): Recently, SMF has been shown to promote the proliferation, migration, and differentiation of stem cells. Furthermore, SMF at 1 mT has been reported to increase DPSC proliferation, and the gene expression of fibroblast growth factor (FGF)-2, TGF-β, and vascular endothelial growth factor (*VEGF*) by upregulating *MMP-1* and *MMP-2* gene expression. SMF also inhibits the phosphorylation of YAP/TAZ, which continuously induces odontoblast differentiation and mineralization in DPSCs.^161^

Electro filed (EF): A potential method (frequency: 1 Hz, 40 ms pulse length and 70 V) using a pulse EF to deliver growth/differentiation factor 11(GDF11) could promote DPSC differentiation into odontoblasts and induce the expression of dentin sialoprotein (Dsp), a differentiation marker for odontoblasts, in the future. This study suggests that the co-application of physical and gene therapy may achieve the goal of dental tissue repair.^162^

Laser irradiation: Low-level laser irradiation (InGaAsP; 940 nm; 0.2 W, continuous mode) at 8 J/cm^2^ stimulated cellular proliferation and promoted biomineralization of stem cells from human exfoliated deciduous teeth by upregulating odontogenesis-related genes (DSPP, ALP, BMP-2) .^163^ The development of genomics, molecular biology, biophysics, and materials science in the future will provide more alternative and efficient methods for the regeneration and restoration of dentin. Consequently, the “secrets” of dentinogenesis will be gradually unveiled.

## References

[1] Meyle J, Dommisch H, Groeger S, Giacaman RA, Costalonga M, Herzberg M. The innate host response in caries and periodontitis. J Clin Periodontol. 2017;44(12):1215–25. doi:10.1111/jcpe.12781. 28727164

[2] Budsamongkol T, Intarak N, Theerapanon T, Yodsanga S, Porntaveetus T, Shotelersuk V. A novel mutation in col1a2 leads to osteogenesis imperfecta/ehlers-danlos overlap syndrome with brachydactyly. Genes Dis. 2019;6(2):138–46. doi:10.1016/j.gendis.2019.03.001. 31193991 PMC6545454

[3] Andersson K, Dahllöf G, Lindahl K, Kindmark A, Grigelioniene G, Åström E, Malmgren B. Mutations in col1a1 and col1a2 and dental aberrations in children and adolescents with osteogenesis imperfecta–a retrospective cohort study. PLoS One. 2017;12(5):e0176466. doi:10.1371/journal.pone.0176466. 28498836 PMC5428910

[4] Bonaventure J, Stanescu R, Stanescu V, Allain JC, Muriel MP, Ginisty D, Maroteaux P. Type ii collagen defect in two sibs with the goldblatt syndrome, a chondrodysplasia with dentinogenesis imperfecta, and joint laxity. Am J Med Genet. 1992;44(6):738–53. doi:10.1002/ajmg.1320440607. 1481841

[5] Nagai N, Nakano K, Sado Y, Naito I, Gunduz M, Tsujigiwa H, Nagatsuka H, Ninomiya Y, Siar CH. Localization of type iv collagen a 1 to a 6 chains in basement membrane during mouse molar germ development. Int J Dev Biol. 2001;45:827–31. 11732842

[6] Yuan G, Chen L, Feng J, Yang G, Ni Q, Xu X, Wan C, Lindsey M, Donly KJ, MacDougall M. Dentin sialoprotein is a novel substrate of matrix metalloproteinase 9 in vitro and in vivo. Sci Rep. 2017;7:42449. doi:10.1038/srep42449. 28195206 PMC5307955

[7] Duverger O, Zah A, Isaac J, Sun H-W, Bartels AK, Lian JB, Berdal A, Hwang J, Morasso MI. Neural crest deletion of dlx3 leads to major dentin defects through down-regulation of dspp. J Biol Chem. 2012;287(15):12230–40. doi:10.1074/jbc.M111.326900. 22351765 PMC3320974

[8] De Vega S, Iwamoto T, Nakamura T, Hozumi K, McKnight DA, Fisher LW, Fukumoto S, Yamada Y. Tm14 is a new member of the fibulin family (fibulin-7) that interacts with extracellular matrix molecules and is active for cell binding. J Biol Chem. 2007;282(42):30878–88. doi:10.1074/jbc.M705847200. 17699513

[9] Vijaykumar A, Dyrkacz P, Vidovic-Zdrilic I, Maye P, Mina M. Expression of bsp-gfptpz transgene during osteogenesis and reparative dentinogenesis. J Dent Res. 2020;99(1):89–97. doi:10.1177/0022034519885089. 31682548 PMC6927219

[10] Qin C, D’Souza R, Feng J. Dentin matrix protein 1 (dmp1): new and important roles for biomineralization and phosphate homeostasis. J Dent Res. 2007;86(12):1134–41. doi:10.1177/154405910708601202. 18037646

[11] Tompkins K, Alvares K, George A, Veis A. Two related low molecular mass polypeptide isoforms of amelogenin have distinct activities in mouse tooth germ differentiation in vitro. J Bone Miner Res. 2005;20(2):341–49. doi:10.1359/JBMR.041107. 15647828

[12] George A, Srinivasan R, Thotakura SR, Liu K, Veis A. Rat dentin matrix protein 3 is a compound protein of rat dentin sialoprotein and phosphophoryn. Connect Tissue Res. 1999;40(1):49–57. doi:10.3109/03008209909005277. 10770650

[13] Hao J, Narayanan K, Muni T, Ramachandran A, George A. Dentin matrix protein 4, a novel secretory calcium-binding protein that modulates odontoblast differentiation. J Biol Chem. 2007;282(21):15357–65. doi:10.1074/jbc.M701547200. 17369251

[14] Imhof T, Korkmaz Y, Koch M, Sengle G, Schiavinato A. Emilin proteins are novel extracellular constituents of the dentin-pulp complex. Sci Rep. 2020;10(1):15320. doi:10.1038/s41598-020-72123-2. 32948785 PMC7501263

[15] Xiong F, Ji Z, Liu Y, Zhang Y, Hu L, Yang Q, Qiu Q, Zhao L, Chen D, Tian Z. Mutation in ssuh2 causes autosomal‐dominant dentin dysplasia type i. Hum Mutat. 2017;38(1):95–104. doi:10.1002/humu.23130. 27680507

[16] Ravindran S, Narayanan K, Eapen AS, Hao J, Ramachandran A, Blond S, George A. Endoplasmic reticulum chaperone protein grp-78 mediates endocytosis of dentin matrix protein 1. J Biol Chem. 2008;283(44):29658–70. doi:10.1074/jbc.M800786200. 18757373 PMC2573078

[17] Hayano S, Kurosaka H, Yanagita T, Kalus I, Milz F, Ishihara Y, Islam MN, Kawanabe N, Saito M, Kamioka H. Roles of heparan sulfate sulfation in dentinogenesis. J Biol Chem. 2012;287(15):12217–29. doi:10.1074/jbc.M111.332924. 22351753 PMC3320973

[18] Palosaari H, Pennington CJ, Larmas M, Edwards DR, Tjäderhane L, Salo T. Expression profile of matrix metalloproteinases (mmps) and tissue inhibitors of mmps in mature human odontoblasts and pulp tissue. Eur J Oral Sci. 2003;111(2):117–27. doi:10.1034/j.1600-0722.2003.00026.x. 12648263

[19] Küchler EC, Menezes R, Callahan N, Costa MC, Modesto A, Meira R, Patir A, Seymen F, Paiva KB, Nunes FD. Mmp1 and mmp20 contribute to tooth agenesis in humans. Arch Oral Biol. 2011;56(5):506–11. doi:10.1016/j.archoralbio.2010.11.007. 21144496

[20] Xu H, Snider T, Wimer H, Yamada S, Yang T, Holmbeck K, Foster B. Multiple essential mt1-mmp functions in tooth root formation, dentinogenesis, and tooth eruption. Matrix Biol. 2016;52:266–83. doi:10.1016/j.matbio.2016.01.002. 26780723 PMC4875876

[21] Yoshiba N, Yoshiba K, Stoetzel C, Perrin‐Schmitt F, Cam Y, Ruch JV, Lesot H. Temporospatial gene expression and protein localization of matrix metalloproteinases and their inhibitors during mouse molar tooth development. Dev Dyn. 2003;228(1):105–12. doi:10.1002/dvdy.10352. 12950084

[22] Papagerakis P, Berdal A, Mesbah M, Peuchmaur M, Malaval L, Nydegger J, Simmer J, Macdougall M. Investigation of osteocalcin, osteonectin, and dentin sialophosphoprotein in developing human teeth. Bone. 2002;30(2):377–85. doi:10.1016/S8756-3282(01)00683-4. 11856645

[23] Gullard A, Gluhak-Heinrich J, Papagerakis S, Sohn P, Unterbrink A, Chen S, MacDougall M. Mepe localization in the craniofacial complex and function in tooth dentin formation. J Histochem Cytochem. 2016;64(4):224–36. doi:10.1369/0022155416635569. 26927967 PMC4817730

[24] Lv H, Fu S, Wu G, Yan F. Phex neutralizing agent inhibits dentin formation in mouse tooth germ. Tissue Cell. 2011;43(2):125–30. doi:10.1016/j.tice.2010.12.008. 21324501

[25] Hikake T, Mori T, Iseki K, Hagino S, Zhang Y, Takagi H, Yokoya S, Wanaka A. Comparison of expression patterns between creb family transcription factor oasis and proteoglycan core protein genes during murine tooth development. Anat Embryol (Berl). 2003;206(5):373–80. doi:10.1007/s00429-003-0311-z. 12684764

[26] Thesleff I, Mackie E, Vainio S, Chiquet-Ehrismann R. Changes in the distribution of tenascin during tooth development. Development. 1987;101(2):289–96. doi:10.1242/dev.101.2.289. 2451586

[27] Zeichner-David M, Vo H, Tan H, Diekwisch T, Berman B, Thiemann F, Alcocer M, Hsu P, Wang T, Eyna J. Timing of the expression of enamel gene products during mouse tooth development. Int J Dev Biol. 2003;41:27–38. 9074935

[28] Heymann R, Kallenbach S, Alonso S, Carroll P, Mitsiadis TA. Dynamic expression patterns of the new protocadherin families cnrs and pcdh-γ during mouse odontogenesis: comparison with reelin expression. Mech Dev. 2001;106(1–2):181–84. doi:10.1016/S0925-4773(01)00433-6. 11472853

[29] Maurin J-C, Couble M-L, Didier-Bazes M, Brisson C, Magloire H, Bleicher F. Expression and localization of reelin in human odontoblasts. Matrix Biol. 2004;23(5):277–85. doi:10.1016/j.matbio.2004.06.005. 15464360

[30] Komichi S, Takahashi Y, Okamoto M, Ali M, Watanabe M, Huang H, Nakai T, Cooper P, Hayashi M. Protein s100-a7 derived from digested dentin is a critical molecule for dentin pulp regeneration. Cells. 2019;8(9). doi:10.3390/cells8091002. PMC6769619 31470634

[31] James MJ, Järvinen E, Thesleff I. Bono1: a gene associated with regions of deposition of bone and dentine. Gene Expression Patterns. 2004;4(5):595–99. doi:10.1016/j.modgep.2004.01.013. 15261838

[32] Takeuchi R, Ohkura N, Yoshiba K, Tohma A, Yoshiba N, Edanami N, Shirakashi M, Belal RSI, Ohshima H, Noiri Y. Immunohistochemistry and gene expression of glut1, runx2 and mtor in reparative dentinogenesis. Oral Dis. 2020;26(2):341–49. doi:10.1111/odi.13230. 31710760

[33] Bidder M, Latifi T, Towler DA. Reciprocal temporospatial patterns of msx2 and osteocalcin gene expression during murine odontogenesis. J Bone Miner Res. 1998;13(4):609–19. doi:10.1359/jbmr.1998.13.4.609. 9556061

[34] Wang S-K, Chan H-C, Makovey I, Simmer JP, Hu JC. Novel pax9 and col1a2 missense mutations causing tooth agenesis and oi/dgi without skeletal abnormalities. PLoS One. 2012;7(12):e51533. doi:10.1371/journal.pone.0051533. 23227268 PMC3515487

[35] Saito K, Nakatomi M, Ida-Yonemochi H, Ohshima H. Osteopontin is essential for type i collagen secretion in reparative dentin. J Dent Res. 2016;95(9):1034–41. doi:10.1177/0022034516645333. 27126446

[36] Zhang X, Rahemtulla FG, MacDougall MJ, Thomas HF. Vitamin d receptor deficiency affects dentin maturation in mice. Arch Oral Biol. 2007;52(12):1172–79. doi:10.1016/j.archoralbio.2007.06.010. 17707333

[37] Chae Y-M, Heo S-H, Kim J-Y, Lee J-M, Ryoo H-M, Cho J-Y. Upregulation of smpd3 via bmp2 stimulation and runx2. BMB Reports. 2009;42(2):86–90. doi:10.5483/BMBRep.2009.42.2.086. 19250608

[38] Petersson U, Hultenby K, Wendel M. Identification, distribution and expression of osteoadherin during tooth formation. Eur J Oral Sci. 2003;111(2):128–36. doi:10.1034/j.1600-0722.2003.00027.x. 12648264

[39] Collignon A-M, Amri N, Lesieur J, Sadoine J, Ribes S, Menashi S, Simon S, Berdal A, Rochefort G, Chaussain C. Sclerostin deficiency promotes reparative dentinogenesis. J Dent Res. 2017;96(7):815–21. doi:10.1177/0022034517698104. 28571484

[40] Ao M, Chavez M, Chu E, Hemstreet K, Yin Y, Yadav M, Millán J, Fisher L, Goldberg H, Somerman M. Overlapping functions of bone sialoprotein and pyrophosphate regulators in directing cementogenesis. Bone. 2017;105:134–47. doi:10.1016/j.bone.2017.08.027. 28866368 PMC5730356

[41] Chen S, Gluhak-Heinrich J, Wang Y, Wu Y, Chuang H, Chen L, Yuan G, Dong J, Gay I, MacDougall M. Runx2, osx, and dspp in tooth development. J Dent Res. 2009;88(10):904–09. doi:10.1177/0022034509342873. 19783797 PMC3045537

[42] Morkmued S, Clauss F, Schuhbaur B, Fraulob V, Mathieu E, Hemmerlé J, Clevers H, Koo B-K, Dollé P, Bloch-Zupan A. Deficiency of the smoc2 matricellular protein impairs bone healing and produces age-dependent bone loss. Sci Rep. 2020;10(1):1–14. doi:10.1038/s41598-020-71749-6. 32908163 PMC7481257

[43] Muromachi K, Kamio N, Matsuki‐Fukushima M, Nishimura H, Tani‐Ishii N, Sugiya H, Matsushima K. Ccn 2/ctgf expression via cellular uptake of bmp‐1 is associated with reparative dentinogenesis. Oral Dis. 2015;21(6):778–84. doi:10.1111/odi.12347. 25944709

[44] Akamatsu T, Matsuda Y, Tsumura K, Tada J, Parvin MN, Wei W, Kanamori N, Hosoi K. Highly regulated expression of subtilisin-like proprotein convertase pace4 (spc4) during dentinogenesis. Biochem Biophys Res Commun. 2000;272(2):410–15. doi:10.1006/bbrc.2000.2752. 10833428

[45] Mao X, Liu S, Lin Y, Chen Z, Shao Y, Yu Q, Liu H, Lu Z, Sheng H, Lu X. Two novel mutations in the alpl gene of unrelated Chinese children with hypophosphatasia: case reports and literature review. BMC Pediatr. 2019;19(1):456. doi:10.1186/s12887-019-1800-4. 31760938 PMC6876108

[46] Ohazama A, Courtney J-M, Sharpe P. Opg, rank, and rankl in tooth development: co-ordination of odontogenesis and osteogenesis. J Dent Res. 2004;83(3):241–44. doi:10.1177/154405910408300311. 14981127

[47] Yoshioka S, Takahashi Y, Abe M, Michikami I, Imazato S, Wakisaka S, Hayashi M, Ebisu S. Activation of the wnt/β-catenin pathway and tissue inhibitor of metalloprotease 1 during tertiary dentinogenesis. J Biochem. 2013;153(1):43–50. doi:10.1093/jb/mvs117. 23038674 PMC3527996

[48] Mostowska A, Biedziak B, Zadurska M, Dunin‐Wilczynska I, Lianeri M, Jagodzinski P. Nucleotide variants of genes encoding components of the wnt signalling pathway and the risk of non‐syndromic tooth agenesis. Clin Genet. 2013;84(5):429–40. doi:10.1111/cge.12061. 23167694

[49] Yu F, Cai W, Jiang B, Xu L, Liu S, Zhao S. A novel mutation of adenomatous polyposis coli (apc) gene results in the formation of supernumerary teeth. J Cell Mol Med. 2018;22(1):152–62. doi:10.1111/jcmm.13303. 28782241 PMC5742724

[50] Issa YA, Kamal L, Rayyan AA, Dweik D, Pierce S, Lee MK, King M-C, Walsh T, Kanaan M. Mutation of kremen1, a modulator of wnt signaling, is responsible for ectodermal dysplasia including oligodontia in Palestinian families. Eur J Human Genet. 2016;24(10):1430–35. doi:10.1038/ejhg.2016.29. 27049303 PMC5027678

[51] Ockeloen CW, Khandelwal KD, Dreesen K, Ludwig KU, Sullivan R, Van Rooij IA, Thonissen M, Swinnen S, Phan M, Conte F. Novel mutations in lrp6 highlight the role of wnt signaling in tooth agenesis. Genet Med. 2016;18(11):1158. doi:10.1038/gim.2016.10. 26963285 PMC5018235

[52] Lammi L, Arte S, Somer M, Jarvinen H, Lahermo P, Thesleff I, Pirinen S, Nieminen P. Mutations in axin2 cause familial tooth agenesis and predispose to colorectal cancer. Am J Hum Genet. 2004;74(5):1043–50. doi:10.1086/386293. 15042511 PMC1181967

[53] Beck DB, Subramanian T, Vijayalingam S, Ezekiel UR, Donkervoort S, Yang ML, Dubbs HA, Ortiz-Gonzalez XR, Lakhani S, Segal D. A pathogenic ctbp1 missense mutation causes altered cofactor binding and transcriptional activity. neurogenetics. 2019;20(3):129–43. doi:10.1007/s10048-019-00578-1. 31041561 PMC8078134

[54] Yu G, Wang J, Lin X, Diao S, Cao Y, Dong R, Wang L, Wang S, Fan Z. Demethylation of sfrp 2 by histone demethylase kdm 2a regulated osteo‐/dentinogenic differentiation of stem cells of the apical papilla. Cell Prolif. 2016;49(3):330–40. doi:10.1111/cpr.12256. 27074224 PMC6496193

[55] Nakatomi M, Ida-Yonemochi H, Ohshima H. Lymphoid enhancer-binding factor 1 expression precedes dentin sialophosphoprotein expression during rat odontoblast differentiation and regeneration. J Endod. 2013;39(5):612–18. doi:10.1016/j.joen.2012.12.016. 23611378

[56] Yamashiro T, Zheng L, Shitaku Y, Saito M, Tsubakimoto T, Takada K, Takano‐Yamamoto T, Thesleff I. Wnt10a regulates dentin sialophosphoprotein mrna expression and possibly links odontoblast differentiation and tooth morphogenesis. Differentiation. 2007;75(5):452–62. doi:10.1111/j.1432-0436.2006.00150.x. 17286598

[57] Kantaputra P, Hutsadaloi A, Kaewgahya M, Intachai W, German R, Koparal M, Leethanakul C, Tolun A, Ketudat Cairns J. Wnt10b mutations associated with isolated dental anomalies. Clin Genet. 2018;93(5):992–99. doi:10.1111/cge.13218. 29364501

[58] Chen D, Yu F, Wu F, Bai M, Lou F, Liao X, Wang C, Ye L. The role of wnt7b in the mediation of dentinogenesis via the erk1/2 pathway. Arch Oral Biol. 2019;104:123–32. doi:10.1016/j.archoralbio.2019.05.009. 31181411

[59] Zeng Y, Baugh E, Akyalcin S, Letra A. Functional effects of wnt10a rare variants associated with tooth agenesis. J Dent Res. 2020;22034520962728. 10.1177/0022034520962728 33034246

[60] Pan Y, Lu T, Peng L, Chen Z, Li M, Zhang K, Xiong F, Wu B. Vacuolar protein sorting 4b regulates the proliferation and odontoblastic differentiation of human dental pulp stem cells through the wnt-β-catenin signalling pathway. Artif Cells, Nanomed Biotechnol. 2019;47(1):2575–84. doi:10.1080/21691401.2019.1629950. 31218890

[61] Li J, Xu J, Cui Y, Wang L, Wang B, Wang Q, Zhang X, Qiu M, Zhang Z. Mesenchymal sufu regulates development of mandibular molars via shh signaling. J Dent Res. 2019;98(12):1348–56. doi:10.1177/0022034519872679. 31499014

[62] Binder M, Chmielarz P, Mckinnon PJ, Biggs LC, Thesleff I, Balic A. Functionally distinctive ptch receptors establish multimodal hedgehog signaling in the tooth epithelial stem cell niche. Stem Cells. 2019;37(9):1238–48. doi:10.1002/stem.3042. 31145830 PMC7859980

[63] Zhang Y, Zhao X, Hu Y, St. Amand T, Zhang M, Ramamurthy R, Qiu M, Chen Y. Msx1 is required for the induction of patched by sonic hedgehog in the mammalian tooth germ. Dev Dyn. 1999;215(1):45–53. doi:10.1002/(SICI)1097-0177(199905)215:1<45::AID-DVDY5>3.0.CO;2-5. 10340755

[64] Hosoya A, Shalehin N, Takebe H, Shimo T, Irie K. Sonic hedgehog signaling and tooth development. Int J Mol Sci. 2020;21(5):1587. doi:10.3390/ijms21051587. PMC7084732 32111038

[65] Hardcastle Z, Mo R, Hui C, Sharpe PT. The shh signalling pathway in tooth development: defects in gli2 and gli3 mutants. Development. 1998;125(15):2803–11. doi:10.1242/dev.125.15.2803. 9655803

[66] Roh SY, Park J-C. The role of nuclear factor ic in tooth and bone development. J Korean Assoc Oral Maxillofac Surg. 2017;43(2):63–69. doi:10.5125/jkaoms.2017.43.2.63. 28462188 PMC5410429

[67] Liu B, Chen S, Cheng D, Jing W, Helms J. Primary cilia integrate hedgehog and wnt signaling during tooth development. J Dent Res. 2014;93(5):475–82. doi:10.1177/0022034514528211. 24659776 PMC3988628

[68] Ahn Y, Kim T, Choi H, Bae C, Yang Y, Baek J, Lee J, Cho E. Disruption of tgfbr2 in odontoblasts leads to aberrant pulp calcification. J Dent Res. 2015;94(6):828–35. doi:10.1177/0022034515577427. 25818583

[69] Wang X-P, Suomalainen M, Jorgez CJ, Matzuk MM, Werner S, Thesleff I. Follistatin regulates enamel patterning in mouse incisors by asymmetrically inhibiting bmp signaling and ameloblast differentiation. Dev Cell. 2004;7(5):719–30. doi:10.1016/j.devcel.2004.09.012. 15525533

[70] Heikinheimo K, Begue-Kirn C, Ritvos O, Tuuri T, Ruch J. The activin-binding protein follistatin is expressed in developing murine molar and induces odontoblast-like cell differentiation in vitro. J Dent Res. 1997;76(10):1625–36. doi:10.1177/00220345970760100301. 9326894

[71] Mitsiadis TA, Angeli I, James C, Lendahl U, Sharpe PT. Role of islet1 in the patterning of murine dentition. Development. 2003;130(18):4451–60. doi:10.1242/dev.00631. 12900460

[72] Laurikkala J, Kassai Y, Pakkasjärvi L, Thesleff I, Itoh N. Identification of a secreted bmp antagonist, ectodin, integrating bmp, fgf, and shh signals from the tooth enamel knot. Dev Biol. 2003;264(1):91–105. doi:10.1016/j.ydbio.2003.08.011. 14623234

[73] Yamashiro T, Tummers M, Thesleff I. Expression of bone morphogenetic proteins and msx genes during root formation. J Dent Res. 2003;82(3):172–76. doi:10.1177/154405910308200305. 12598544

[74] Åberg T, Wozney J, Thesleff I. Expression patterns of bone morphogenetic proteins (bmps) in the developing mouse tooth suggest roles in morphogenesis and cell differentiation. Dev Dyn. 1997;210(4):383–96. doi:10.1002/(SICI)1097-0177(199712)210:4<383::AID-AJA3>3.0.CO;2-C. 9415424

[75] Li X, Wang L, Su Q, Ye L, Zhou X, Zhang L, Song D, Huang D. Potential roles of bone morphogenetic protein 9 in the odontogenic differentiation of dental pulp cells. J Endod. 2021;47(3):436–43. doi:10.1016/j.joen.2020.10.018. 33129897

[76] Tao H, Li Q, Lin Y, Zuo H, Cui Y, Chen S, Chen Z, Liu H. Coordinated expression of p300 and hdac3 upregulates histone acetylation during dentinogenesis. J Cell Biochem. 2020;121(3):2478–88. doi:10.1002/jcb.29470. 31692090 PMC7808212

[77] Mitsiadis TA, Catón J, Pagella P, Orsini G, Jimenez-Rojo L. Monitoring notch signaling-associated activation of stem cell niches within injured dental pulp. Front Physiol. 2017;8(372). doi:10.3389/fphys.2017.00372. PMC5447770 28611689

[78] Peled A, Sarig O, Samuelov L, Bertolini M, Ziv L, Weissglas-Volkov D, Eskin-Schwartz M, Adase CA, Malchin N, Bochner R. Mutations in tspear, encoding a regulator of notch signaling, affect tooth and hair follicle morphogenesis. PLoS Genet. 2016;12(10):e1006369. doi:10.1371/journal.pgen.1006369. 27736875 PMC5065119

[79] Qi S, Yan Y, Wen Y, Li J, Wang J, Chen F, Tang X, Shang G, Xu Y, Wang R. The effect of delta‐like 1 homologue on the proliferation and odontoblastic differentiation in human dental pulp stem cells. Cell Prolif. 2017;50(3):e12335. doi:10.1111/cpr.12335. PMC6529117 28205268

[80] Cluzeau C, Hadj‐Rabia S, Jambou M, Mansour S, Guigue P, Masmoudi S, Bal E, Chassaing N, Vincent MC, Viot G. Only four genes (eda1, edar, edaradd, and wnt10a) account for 90% of hypohidrotic/anhidrotic ectodermal dysplasia cases. Hum Mutat. 2011;32(1):70–72. doi:10.1002/humu.21384. 20979233

[81] Ohazama A, Courtney JM, Tucker AS, Naito A, Tanaka S, Inoue JI, Sharpe PT. Traf6 is essential for murine tooth cusp morphogenesis. Dev Dyn. 2004;229(1):131–35. doi:10.1002/dvdy.10400. 14699584

[82] Pispa J, Mikkola ML, Mustonen T, Thesleff I. Ectodysplasin, edar and tnfrsf19 are expressed in complementary and overlapping patterns during mouse embryogenesis. Gene Expression Patterns. 2003;3(5):675–79. doi:10.1016/S1567-133X(03)00092-9. 12972005

[83] Byers MR, Westenbroek RE. Odontoblasts in developing, mature and ageing rat teeth have multiple phenotypes that variably express all nine voltage-gated sodium channels. Arch Oral Biol. 2011;56(11):1199–220. doi:10.1016/j.archoralbio.2011.04.014. 21640979 PMC12028083

[84] Yang J, Cai W, Lu X, Liu S, Zhao S. Rna-sequencing analyses demonstrate the involvement of canonical transient receptor potential channels in rat tooth germ development. Front Physiol. 2017;8(455). doi:10.3389/fphys.2017.00455. PMC5489664 28706494

[85] Hou J, Situ Z, Duan X. Clc chloride channels in tooth germ and odontoblast-like mdpc-23 cells. Arch Oral Biol. 2008;53(9):874–78. doi:10.1016/j.archoralbio.2008.03.009. 18466876

[86] Kwon M, Baek SH, Park C-K, Chung G, Oh SB. Single-cell rt-pcr and immunocytochemical detection of mechanosensitive transient receptor potential channels in acutely isolated rat odontoblasts. Arch Oral Biol. 2014;59(12):1266–71. doi:10.1016/j.archoralbio.2014.07.016. 25150531

[87] Sato M, Ogura K, Kimura M, Nishi K, Ando M, Tazaki M, Shibukawa Y. Activation of mechanosensitive transient receptor potential/piezo channels in odontoblasts generates action potentials in cocultured isolectin b4–negative medium-sized trigeminal ganglion neurons. J Endod. 2018;44(6):984–991. e982. doi:10.1016/j.joen.2018.02.020. 29709295

[88] Lee K, Lee B-M, Park C-K, Kim YH, Chung G. Ion channels involved in tooth pain. Int J Mol Sci. 2019;20(9):2266. doi:10.3390/ijms20092266. PMC6539952 31071917

[89] Wang W, Yi X, Ren Y, Xie Q. Effects of adenosine triphosphate on proliferation and odontoblastic differentiation of human dental pulp cells. J Endod. 2016;42(10):1483–89. doi:10.1016/j.joen.2016.07.013. 27576209

[90] Felszeghy S, Módis L, Németh P, Nagy G, Zelles T, Agre P, Laurikkala J, Fejerskov O, Thesleff I, Nielsen S. Expression of aquaporin isoforms during human and mouse tooth development. Arch Oral Biol. 2004;49(4):247–57. doi:10.1016/j.archoralbio.2003.09.011. 15003543

[91] Gao Y, Sahlberg C, Kiukkonen A, Alaluusua S, Pohjanvirta R, Tuomisto J, Lukinmaa P-L. Lactational exposure of han/wistar rats to 2, 3, 7, 8-tetrachlorodibenzo-p-dioxin interferes with enamel maturation and retards dentin mineralization. J Dent Res. 2004;83(2):139–44. doi:10.1177/154405910408300211. 14742652

[92] Kettunen P, Laurikkala J, Itäranta P, Vainio S, Itoh N, Thesleff I. Associations of fgf‐3 and fgf‐10 with signaling networks regulating tooth morphogenesis. Dev Dyn. 2000;219(3):322–32. doi:10.1002/1097-0177(2000)9999:9999<::AID-DVDY1062>3.0.CO;2-J. 11066089

[93] Kettunen P, Furmanek T, Chaulagain R, Hals Kvinnsland I, Luukko K. Developmentally regulated expression of intracellular fgf11-13, hormone-like fgf15 and canonical fgf16,-17 and-20 mrnas in the developing mouse molar tooth. Acta Odontol Scand. 2011;69(6):360–66. doi:10.3109/00016357.2011.568968. 21449687

[94] Kettunen P, Thesleff I. Expression and function of fgfs-4, −8, and −9 suggest functional redundancy and repetitive use as epithelial signals during tooth morphogenesis. Dev Dyn. 1998;211(3):256–68. doi:10.1002/(SICI)1097-0177(199803)211:3<256::AID-AJA7>3.0.CO;2-G. 9520113

[95] Dos Santos ÍGD, Jorge EC, Copola AGL, Bertassoli BM, de Goes AM, Silva GAB, Dos Santos ÍGD, Goes AMD. Fgf2, fgf3 and fgf4 expression pattern during molars odontogenesis in didelphis albiventris. Acta Histochem. 2017;119(2):129–41. doi:10.1016/j.acthis.2016.12.001. 28012573

[96] Bei M, Maas R. Fgfs and bmp4 induce both msx1-independent and msx1-dependent signaling pathways in early tooth development. Development. 1998;125(21):4325–33. doi:10.1242/dev.125.21.4325. 9753686

[97] Hur S-W, Oh S-H, Jeong B-C, Choi H, Kim J-W, Lee K-N, Hwang Y-C, Ryu J-H, Kim S-H, Koh J-T. Coup-tfii stimulates dentin sialophosphoprotein expression and mineralization in odontoblasts. J Dent Res. 2015;94(8):1135–42. doi:10.1177/0022034515585125. 25940145

[98] Gu J, Ikeda H, Suda H. Sympathetic regulation of tertiary dentinogenesis via beta-2 adrenergic receptor on rat odontoblasts. J Endod. 2015;41(7):1056–60. doi:10.1016/j.joen.2015.01.010. 25702857

[99] Wysolmerski JJ, Cormier S, Philbrick WM, Dann P, Zhang J-P, Roume J, Delezoide A-L, Silve C. Absence of functional type 1 parathyroid hormone (pth)/pth-related protein receptors in humans is associated with abnormal breast development and tooth impaction. J Clin Endocrinol Metab. 2001;86:1788–94. 11297619 10.1210/jcem.86.4.7404

[100] Matsumura S, Quispe-Salcedo A, Schiller C, Shin J, Locke B, Yakar S, Shimizu E. Igf-1 mediates ephrinb1 activation in regulating tertiary dentin formation. J Dent Res. 2017;96(10):1153–61. doi:10.1177/0022034517708572. 28489485 PMC5582682

[101] Pääkkönen V, Saraniemi S, Bleicher F, Nevo Z, Tjäderhane L. Exostosin 1 is expressed in human odontoblasts. Arch Oral Biol. 2017;80:175–79. doi:10.1016/j.archoralbio.2017.04.004. 28448806

[102] Kato Y, Yokose S. Oxytocin facilitates dentinogenesis of rat dental pulp cells. J Endod. 2021;47(4):592–99. doi:10.1016/j.joen.2020.12.017. 33422572

[103] Ohshima H, Ajima H, Kawano Y, Nozawa-Inoue K, Wakisaka S, Maeda T. Transient expression of heat shock protein (hsp) 25 in the dental pulp and enamel organ during odontogenesis in the rat incisor. Arch Histol Cytol. 2000;63(4):381–95. doi:10.1679/aohc.63.381. 11073069

[104] Chen T, Liu Z, Sun W, Li J, Liang Y, Yang X, Xu Y, Yu M, Tian W, Chen G. Inhibition of ape1 redox activity promotes odonto/osteogenic differentiation of dental papilla cells. Sci Rep. 2015;5(17483). doi:10.1038/srep17483. PMC4671010 26639148

[105] Matsuishi YI, Kato H, Masuda K, Yamaza H, Hirofuji Y, Sato H, Wada H, Kiyoshima T, Nonaka K. Accelerated dentinogenesis by inhibiting the mitochondrial fission factor, dynamin related protein 1. Biochem Biophys Res Commun. 2018;495(2):1655–60. doi:10.1016/j.bbrc.2017.12.026. 29223396

[106] Couve E, Osorio R, Schmachtenberg O. Mitochondrial autophagy and lipofuscin accumulation in aging odontoblasts. J Dent Res. 2012;91(7):696–701. doi:10.1177/0022034512449347. 22622661

[107] Sehic A, Khuu C, Risnes S, Osmundsen H. Differential gene expression profiling of the molar tooth germ in peroxisome proliferator‐activated receptor‐α (ppar‐α) knockout mouse and in wild‐type mouse: molar tooth phenotype of ppar‐α knockout mouse. Eur J Oral Sci. 2009;117(2):93–104. doi:10.1111/j.1600-0722.2009.00615.x. 19320717

[108] Lee Y-H, Kang Y-M, Heo M-J, Kim G-E, Bhattarai G, Lee N-H, Yu M-K, Yi H-K. The survival role of peroxisome proliferator-activated receptor gamma induces odontoblast differentiation against oxidative stress in human dental pulp cells. J Endod. 2013;39(2):236–41. doi:10.1016/j.joen.2012.11.006. 23321237

[109] Chen H, Kang J, Zhang F, Yan T, Fan W, He H, Huang F. Sirt4 regulates rat dental papilla cell differentiation by promoting mitochondrial functions. Int J Biochem Cell Biol. 2021;134(105962):105962. doi:10.1016/j.biocel.2021.105962. 33636397

[110] De Vicente J, Cabo R, Ciriaco E, Laura R, Naves F, Silos-Santiago I, Vega J. Impaired dental cytodifferentiation in glial cell-line derived growth factor (gdnf) deficient mice. Ann Anat-Anatomischer Anz. 2002;184(1):85–92. doi:10.1016/S0940-9602(02)80041-3. 11878293

[111] Nakatomi M, Quispe-Salcedo A, Sakaguchi M, Ida-Yonemochi H, Okano H, Ohshima H. Nestin expression is differently regulated between odontoblasts and the subodontoblastic layer in mice. Histochem Cell Biol. 2018;149(4):383–91. doi:10.1007/s00418-018-1651-3. 29445893

[112] Fried K, Risling M, Tidcombe H, Gassmann M, Lillesaar C. Expression of erbb3, erbb4, and neuregulin‐1 mrna during tooth development. Dev Dyn. 2002;224(3):356–60. doi:10.1002/dvdy.10114. 12112465

[113] Zhou C, Yang G, Chen M, Wang C, He L, Xiang L, Chen D, Ling J, Mao JJ. Lhx8 mediated wnt and tgfβ pathways in tooth development and regeneration. Biomaterials. 2015;3:35–46. doi:10.1016/j.biomaterials.2015.06.004. PMC4583713 26081866

[114] Hoshino M, Hashimoto S, Muramatsu T, Matsuki M, Ogiuchi H, Shimono M. Claudin rather than occludin is essential for differentiation in rat incisor odontoblasts. Oral Dis. 2008;14(7):606–12. doi:10.1111/j.1601-0825.2007.01427.x. 18208478

[115] Palacios J, Benito N, Berraquero R, Pizarro A, Cano A, Gamallo C. Differential spatiotemporal expression of e-and p-cadherin during mouse tooth development. Int J Dev Biol. 2004;39:663–66. 8619966

[116] Fried K, Mitsiadis T, Guerrier A, Haegerstrand A, Meister B. Combinatorial expression patterns of the connexins 26, 32, and 43 during development, homeostasis, and regeneration of rat teeth. Int J Dev Biol. 2003;40:985–95. 8946246

[117] Yang J, Lu X, Liu S, Zhao S. The involvement of genes related to bile secretion pathway in rat tooth germ development. J Mol Histol. 2020;51(1):99–107. doi:10.1007/s10735-020-09861-0. 32095972

[118] Li G, Liu M, Zhang S, Wan H, Zhang Q, Yue R, Yan X, Wang X, Wang Z, Sun Y. Essential role of ift140 in promoting dentinogenesis. J Dent Res. 2018;97(4):423–31. doi:10.1177/0022034517741283. 29195058

[119] Wang X, Jong G, Lin LM, Shimizu E. Ephb–ephrinb interaction controls odontogenic/osteogenic differentiation with calcium hydroxide. J Endod. 2013;39(10):1256–60. doi:10.1016/j.joen.2013.06.016. 24041387

[120] Wang L, Yang H, Lin X, Cao Y, Gao P, Zheng Y, Fan Z. Kdm1a regulated the osteo/dentinogenic differentiation process of the stem cells of the apical papilla via binding with plod2. Cell Prolif. 2018;51(4):e12459. doi:10.1111/cpr.12459. 29656462 PMC6528894

[121] Li QM, Li JL, Feng ZH, Lin HC, Xu Q. Effect of histone demethylase kdm5a on the odontogenic differentiation of human dental pulp cells. Bioengineered. 2020;11(1):449–62. doi:10.1080/21655979.2020.1743536. 32208897 PMC7161540

[122] Schneider K, Korkmaz Y, Addicks K, Lang H, Raab WH-M. Prion protein (prp) in human teeth: an unprecedented pointer to prp’s function. J Endod. 2007;33(2):110–13. doi:10.1016/j.joen.2006.11.010. 17258625

[123] Li X-Y, Ban G-F, Al-Shameri B, He X, Liang D-Z, Chen W-X. High-temperature requirement protein a1 regulates odontoblastic differentiation of dental pulp cells via the transforming growth factor beta 1/smad signaling pathway. J Endod. 2018;44(5):765–72. doi:10.1016/j.joen.2018.02.003. 29580722

[124] Kim J-Y, Kim D-S, Auh Q-S, Yi J-K, Moon SU, Kim E-C. Role of protein phosphatase 1 in angiogenesis and odontoblastic differentiation of human dental pulp cells. J Endod. 2017;43(3):417–24. doi:10.1016/j.joen.2016.10.013. 28231980

[125] Yoshida S, Wada N, Hasegawa D, Miyaji H, Mitarai H, Tomokiyo A, Hamano S, Maeda H. Semaphorin 3a induces odontoblastic phenotype in dental pulp stem cells. J Dent Res. 2016;95(11):1282–90. doi:10.1177/0022034516653085. 27302880

[126] Park S, Lee Y, Lee D, Park J, Kim R, Shon W. Cpne7 induces biological dentin sealing in a dentin hypersensitivity model. J Dent Res. 2019;98(11):1239–44. doi:10.1177/0022034519869577. 31425664

[127] Maurin J-C, Couble M-L, Staquet M-J, Carrouel F, About I, Avila J, Magloire H, Bleicher F. Microtubule-associated protein 1b, a neuronal marker involved in odontoblast differentiation. J Endod. 2009;35(7):992–96. doi:10.1016/j.joen.2009.04.009. 19567321

[128] McKee M, Yadav M, Foster B, Somerman M, Farquharson C, Millán J. Compounded phospho1/alpl deficiencies reduce dentin mineralization. J Dent Res. 2013;92(8):721–27. doi:10.1177/0022034513490958. 23694930 PMC3711567

[129] Park Y, Lee Y, Seo Y, Seo H, Park J, Bae H, Park J. Midkine promotes odontoblast-like differentiation and tertiary dentin formation. J Dent Res. 2020;0022034520925427. 10.1177/0022034520925427 32442055

[130] Zheng J, Nie X, He L, Yoon A, Wu L, Zhang X, Vats M, Schiff M, Xiang L, Tian Z. Epithelial cdc42 deletion induced enamel organ defects and cystogenesis. J Dent Res. 2018;97(12):1346–54. doi:10.1177/0022034518779546. 29874522 PMC6199676

[131] Nakamura T, Unda F, De-Vega S, Vilaxa A, Fukumoto S, Yamada KM, Yamada Y. The krüppel-like factor epiprofin is expressed by epithelium of developing teeth, hair follicles, and limb buds and promotes cell proliferation. J Biol Chem. 2004;279(1):626–34. doi:10.1074/jbc.M307502200. 14551215

[132] Bouwman P, Göllner H, Elsässer HP, Eckhoff G, Karis A, Grosveld F, Philipsen S, Suske G. Transcription factor sp3 is essential for post‐natal survival and late tooth development. EMBO J. 2000;19(4):655–61. doi:10.1093/emboj/19.4.655. 10675334 PMC305603

[133] Jimenez-Rojo L, Ibarretxe G, Aurrekoetxea M, de Vega S, Nakamura T, Yamada Y, Unda F. Epiprofin-sp6. A new player in the regulation of tooth development. Histol Histopathol. 2010;25(12):1621–30. doi:10.14670/HH-25.1621. 20886441

[134] Bae JM, Clarke JC, Rashid H, Adhami MD, McCullough K, Scott JS, Chen H, Sinha KM, de Crombrugghe B, Javed A. Specificity protein 7 is required for proliferation and differentiation of ameloblasts and odontoblasts. J Bone Miner Res. 2018;33(6):1126–40. doi:10.1002/jbmr.3401. 29405385 PMC6002875

[135] Xiao Y, Lin YX, Cui Y, Zhang Q, Pei F, Zuo HY, Liu H, Chen Z. Zeb1 promotes odontoblast differentiation in a stage-dependent manner. J Dent Res. 2021;22034520982249. 10.1177/0022034520982249 33419386

[136] Aryal YP, Neupane S, Kim TY, Lee ES, Pokhrel NK, Yeon CY, Kim JY, An CH, An SY, Park EK, et al. Developmental roles of fuse binding protein 1 (fubp1) in tooth morphogenesis. Int J Mol Sci. 2020;21(21):8079. doi:10.3390/ijms21218079. PMC7663687 33138041

[137] Zhang Y, Fang M, Yang Z, Qin W, Guo S, Ma J, Chen W. Gata binding protein 4 regulates tooth root dentin development via fbp1. Int J Biol Sci. 2020;16(1):181–93. doi:10.7150/ijbs.36567. 31892855 PMC6930368

[138] De Coster P, Cornelissen M, De Paepe A, Martens L, Vral A. Abnormal dentin structure in two novel gene mutations [col1a1, arg134cys] and [adamts2, trp795-to-ter] causing rare type i collagen disorders. Arch Oral Biol. 2007;52(2):101–09. doi:10.1016/j.archoralbio.2006.08.007. 17118335

[139] Goss M, Socorro M, Monier D, Verdelis K, Napierala D. Trps1 transcription factor regulates mineralization of dental tissues and proliferation of tooth organ cells. Mol Genet Metab. 2019;126(4):504–12. doi:10.1016/j.ymgme.2019.01.014. 30691926 PMC6535116

[140] Abe M, Tamamura Y, Yamagishi H, Maeda T, Kato J, Tabata MJ, Srivastava D, Wakisaka S, Kurisu K. Tooth-type specific expression of dhand/hand2: possible involvement in murine lower incisor morphogenesis. Cell Tissue Res. 2002;310(2):201–12. doi:10.1007/s00441-002-0611-2. 12397375

[141] Zhang Y, Lian M, Zhao X, Cao P, Xiao J, Shen S, Tang W, Zhang J, Hao J, Feng X. Rick regulates the odontogenic differentiation of dental pulp stem cells through activation of tnf-alpha via the erk and not through nf-kappab signaling pathway. Cell Biol Int. 2020. 10.1002/cbin.11498 33169892

[142] Fu J, Zheng H, Xue Y, Jin R, Yang G, Chen Z, Yuan G. Wwp2 promotes odontoblastic differentiation by monoubiquitinating klf5. J Dent Res. 2020;22034520970866. 10.1177/0022034520970866 33164644

[143] Lekszas C, Foresti O, Raote I, Liedtke D, Konig EM, Nanda I, Vona B, De Coster P, Cauwels R, Malhotra V, et al. Biallelic tango1 mutations cause a novel syndromal disease due to hampered cellular collagen secretion. Elife. 2020;9. doi:10.7554/eLife.51319. PMC7062462 32101163

[144] Tohma A, Ohkura N, Yoshiba K, Takeuchi R, Yoshiba N, Edanami N, Shirakashi M, Ibn Belal RS, Ohshima H, Noiri Y. Glucose transporter 2 and 4 are involved in glucose supply during pulpal wound healing after pulpotomy with mineral trioxide aggregate in rat molars. J Endod. 2020;46(1):81–88. doi:10.1016/j.joen.2019.10.003. 31767340

[145] Yan W, Cao Y, Yang H, Han N, Zhu X, Fan Z, Du J, Zhang F. Cb1 enhanced the osteo/dentinogenic differentiation ability of periodontal ligament stem cells via p38 mapk and jnk in an inflammatory environment. Cell Prolif. 2019;52(6):e12691. doi:10.1111/cpr.12691. 31599069 PMC6869632

[146] Fujihara H, Nozaki T, Tsutsumi M, Isumi M, Shimoda S, Hamada Y, Masutani M. Spontaneous development of dental dysplasia in aged parp-1 knockout mice. Cells. 2019;8(10). doi:10.3390/cells8101157. PMC6829344 31569682

[147] Jiang S, Sheng R, Qi X, Wang J, Guo Y, Yuan Q. Usp34 regulates tooth root morphogenesis by stabilizing nfic. Int J Oral Sci. 2021;13(1):7. doi:10.1038/s41368-021-00114-8. 33686052 PMC7940473

[148] Luo X, Yin J, Miao S, Feng W, Ning T, Xu S, Huang S, Zhang S, Liao Y, Hao C, et al. Mtorc1 promotes mineralization via p53 pathway. FASEB J. 2021;35(2):e21325. doi:10.1096/fj.202002016R. 33508145

[149] Sulkala M, Larmas M, Sorsa T, Salo T, Tjaderhane L. The localization of matrix metalloproteinase-20 (mmp-20, enamelysin) in mature human teeth. J Dent Res. 2002;81(9):603–07. doi:10.1177/154405910208100905. 12202640

[150] Wang DY, Zhang L, Fan J, Li F, Ma KQ, Wang P, Chen JH. Matrix metalloproteinases in human sclerotic dentine of attrited molars. Arch Oral Biol. 2012;57(10):1307–12. doi:10.1016/j.archoralbio.2012.04.012. 22608916

[151] Koyama E, Wu C, Shimo T, Iwamoto M, Ohmori T, Kurisu K, Ookura T, Bashir MM, Abrams WR, Tucker T, et al. Development of stratum intermedium and its role as a sonic hedgehog-signaling structure during odontogenesis. Dev Dyn. 2001;222(2):178–91. doi:10.1002/dvdy.1186. 11668596

[152] Wang S, Mu J, Fan Z, Yu Y, Yan M, Lei G, Tang C, Wang Z, Zheng Y, Yu J, et al. Insulin-like growth factor 1 can promote the osteogenic differentiation and osteogenesis of stem cells from apical papilla. Stem Cell Res. 2012;8(3):346–56. doi:10.1016/j.scr.2011.12.005. 22286010

[153] Ohshima H, Ajima H, Kawano Y, Nozawa-Inoue K, Wakisaka S, Maeda T. Transient expression of heat shock protein (hsp)25 in the dental pulp and enamel organ during odontogenesis in the rat incisor. Arch Histol Cytol. 2000;63(4):381–95. doi:10.1679/aohc.63.381. 11073069

[154] Couve E, Osorio R, Schmachtenberg O. The amazing odontoblast: activity, autophagy, and aging. J Dent Res. 2013;92(9):765–72. doi:10.1177/0022034513495874. 23803461

[155] Couve E, Schmachtenberg O. Autophagic activity and aging in human odontoblasts. J Dent Res. 2011;90(4):523–28. doi:10.1177/0022034510393347. 21212314

[156] Arola D, Reprogel R. Effects of aging on the mechanical behavior of human dentin. Biomaterials. 2005;26(18):4051–61. doi:10.1016/j.biomaterials.2004.10.029. 15626451

[157] Tagami J, Hosoda H, Burrow M, Nakajima M. Effect of aging and caries on dentin permeability. Proceedings of the Finnish Dental Society. Suomen Hammaslaakariseuran toimituksia. Vol. 88; 1992. p. 149–54. 1508869

[158] Robertson KD, Wolffe AP. DNA methylation in health and disease. Nat Rev Genet. 2000;1(1):11–19. doi:10.1038/35049533. 11262868

[159] Xuan K, Li B, Guo H, Sun W, Kou X, He X, Zhang Y, Sun J, Liu A, Liao L. Deciduous autologous tooth stem cells regenerate dental pulp after implantation into injured teeth. Sci Transl Med. 2018;10(455). doi:10.1126/scitranslmed.aaf3227. 30135248

[160] Zuo J, Zhen J, Wang F, Li Y, Zhou Z. Effect of low-intensity pulsed ultrasound on the expression of calcium ion transport-related proteins during tertiary dentin formation. Ultrasound Med Biol. 2018;44(1):223–33. doi:10.1016/j.ultrasmedbio.2017.09.006. 29079395

[161] Zheng L, Zhang L, Chen L, Jiang J, Zhou X, Wang M, Fan Y. Static magnetic field regulates proliferation, migration, differentiation, and yap/taz activation of human dental pulp stem cells. J Tissue Eng Regen Med. 2018;12(10):2029–40. doi:10.1002/term.2737. 30058115

[162] Nakashima M, Mizunuma K, Murakami T, Akamine A. Induction of dental pulp stem cell differentiation into odontoblasts by electroporation-mediated gene delivery of growth/differentiation factor 11 (gdf11). Gene Ther. 2002;9(12):814–18. doi:10.1038/sj.gt.3301692. 12040463

[163] Paschalidou M, Athanasiadou E, Arapostathis K, Kotsanos N, Koidis P, Bakopoulou A, Theocharidou A. Biological effects of low-level laser irradiation (llli) on stem cells from human exfoliated deciduous teeth (shed). Clin Oral Investig. 2020;24(1):167–80. doi:10.1007/s00784-019-02874-4. 31069538

